# Bis[4-(4-chloro­phen­yl)-4-hydroxy­piperidinium] dipicrate dimethyl sulfoxide solvate

**DOI:** 10.1107/S1600536810015187

**Published:** 2010-04-30

**Authors:** Hoong-Kun Fun, Madhukar Hemamalini, B. P. Siddaraju, H. S. Yathirajan, B. Narayana

**Affiliations:** a X-ray Crystallography Unit, School of Physics, Universiti Sains Malaysia, 11800 USM, Penang, Malaysia; b Department of Chemistry, V. V. Puram College of Science, Bangalore-560 004, India; c Department of Studies in Chemistry, University of Mysore, Manasagangotri, Mysore 570 006, India; d Department of Studies in Chemistry, Mangalore University, Mangalagangotri 574 199, India

## Abstract

The asymmetric unit of the title salt solvate, 2C_11_H_15_ClNO^+^·2C_6_H_2_N_3_O_7_^−^·C_2_H_6_OS, contains two crystallographically independent 4-(4-chloro­phen­yl)-4-hydroxy­piperidinium cations, two picrate anions and a dimethyl sulfoxide solvent mol­ecule. In each cation, the piperidinium ring adopts a chair conformation. In the crystal structure, the cations, anions and solvent mol­ecules are connected by inter­molecular O—H⋯O, N—H⋯O and C—H⋯O hydrogen bonds, forming a three-dimensional network.

## Related literature

For background to the importance of piperidines, see: Vartanyan (1984 ▶). For related structures, see: Cygler *et al.* (1980 ▶); Cygler & Ahmed (1984 ▶); Dutkiewicz *et al.* (2010 ▶); Georges *et al.* (1989 ▶); Jasinski *et al.* (2009 ▶); Lisgarten & Palmer (1989 ▶); Tomlin *et al.* (1996 ▶). For picrate salts, see: Anitha *et al.* (2004 ▶); Thanigaimani *et al.* (2009 ▶). For ring conformations, see: Cremer & Pople (1975 ▶). For bond-length data, see: Allen *et al.* (1987 ▶). For the stability of the temperature controller used in the data collection, see: Cosier & Glazer (1986 ▶).
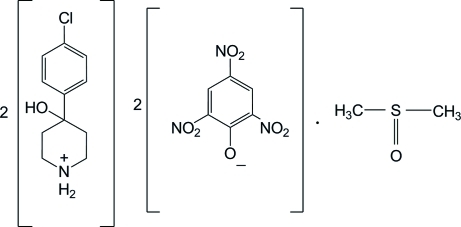


## Experimental

### 

#### Crystal data

2C_11_H_15_ClNO^+^·2C_6_H_2_N_3_O_7_^−^·C_2_H_6_OS
*M* *_r_* = 959.72
Monoclinic, 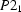

*a* = 8.9207 (4) Å
*b* = 18.1230 (9) Å
*c* = 12.9886 (6) Å
β = 98.430 (1)°
*V* = 2077.18 (17) Å^3^
*Z* = 2
Mo *K*α radiation
μ = 0.29 mm^−1^
*T* = 100 K
0.40 × 0.32 × 0.16 mm

#### Data collection

Bruker APEX DUO CCD area-detector diffractometer
Absorption correction: multi-scan (*SADABS*; Bruker, 2009 ▶) *T*_min_ = 0.893, *T*_max_ = 0.954
25314 measured reflections
15185 independent reflections
13604 reflections with *I* > 2σ(*I*)
*R*_int_ = 0.029

#### Refinement

*R*[*F* ^2^ > 2σ(*F* ^2^)] = 0.041
*wR*(*F* ^2^) = 0.140
*S* = 1.11
15185 reflections
582 parameters
1 restraint
H-atom parameters constrained
Δρ_max_ = 0.83 e Å^−3^
Δρ_min_ = −0.88 e Å^−3^

### 

Data collection: *APEX2* (Bruker, 2009 ▶); cell refinement: *SAINT* (Bruker, 2009 ▶); data reduction: *SAINT*; program(s) used to solve structure: *SHELXTL* (Sheldrick, 2008 ▶); program(s) used to refine structure: *SHELXTL*; molecular graphics: *SHELXTL*; software used to prepare material for publication: *SHELXTL* and *PLATON* (Spek, 2009 ▶).

## Supplementary Material

Crystal structure: contains datablocks global, I. DOI: 10.1107/S1600536810015187/tk2662sup1.cif

Structure factors: contains datablocks I. DOI: 10.1107/S1600536810015187/tk2662Isup2.hkl

Additional supplementary materials:  crystallographic information; 3D view; checkCIF report

## Figures and Tables

**Table 1:** Hydrogen-bond geometry (Å, °)

*D*—H⋯*A*	*D*—H	H⋯*A*	*D*⋯*A*	*D*—H⋯*A*
N1*A*—H1*AB*⋯O7*B*^i^	0.90	2.44	3.030 (2)	123
N1*A*—H1*AB*⋯O8*B*^i^	0.90	2.15	3.048 (2)	176
O1*A*—H1*A*⋯O1*B*^ii^	0.82	2.14	2.8642 (19)	148
O1*B*—H1*B*⋯O9^iii^	0.82	1.83	2.629 (2)	165
N1*A*—H1*AC*⋯O2*A*	0.90	1.83	2.704 (2)	162
N1*A*—H1*AC*⋯O3*A*	0.90	2.30	2.846 (2)	119
N1*B*—H1*BB*⋯O3*A*^iv^	0.90	2.15	3.044 (2)	171
N1*B*—H1*BB*⋯O4*A*^iv^	0.90	2.52	3.101 (2)	123
N1*B*—H1*BC*⋯O2*B*	0.90	1.84	2.714 (2)	162
C4*A*—H4*AA*⋯O4*A*^v^	0.93	2.51	3.311 (2)	145
C5*A*—H5*AA*⋯O1*B*^ii^	0.93	2.52	3.313 (2)	143
C8*A*—H8*AA*⋯O9^vi^	0.97	2.58	3.479 (2)	155
C9*A*—H9*AB*⋯O4*B*^vii^	0.97	2.59	3.469 (3)	151
C11*A*—H11*A*⋯O2*A*	0.97	2.55	3.261 (2)	130
C11*A*—H11*B*⋯O4*B*	0.97	2.59	3.258 (3)	126
C2*B*—H2*BA*⋯O7*B*^v^	0.93	2.60	3.361 (3)	140
C14*B*—H14*B*⋯O6*A*^i^	0.93	2.45	3.335 (3)	160
C5*B*—H5*BA*⋯O3*B*	0.93	2.54	3.424 (2)	160
C16*A*—H16*A*⋯O5*B*^iv^	0.93	2.51	3.418 (3)	166

Symmetry codes: (i) 
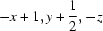
; (ii) 
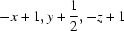
; (iii) 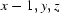; (iv) 
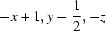
; (v) 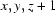; (vi) 
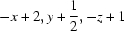
; (vii) 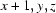.

**Fig. 1.**
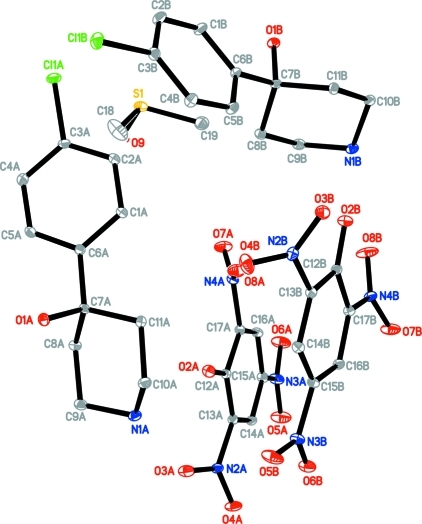
The asymmetric unit of the title compound, showing 30% probability displacement ellipsoids and the atom-numbering scheme. H atoms are omitted for clarity.

**Fig. 2.**
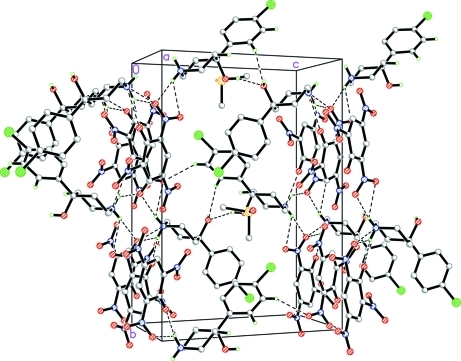
The crystal packing of the title compound, showing hydrogen-bonded (dashed lines) network. H atoms are not involving the hydrogen bond interactions are omitted for clarity.

## supplementary crystallographic information

### Comment

4-(4-Chlorophenyl)-4-hydroxypiperidine is used as an intermediate for the synthesis of pharmaceuticals such as haloperidol (a neuroleptic drug used to treat patients with psychotic illnesses, extreme agitation, or Tourette's syndrome) and loperamide which is a synthetic piperidine derivative and a drug effective against diarrhea resulting from gastroenteritis or inflammatory bowel disease. A review on the synthesis and biological activity of uncondensed cyclic derivatives of piperidine is available (Vartanyan, 1984). The crystal structures of 1,2,2,4,6,6- hexamethyl-4-piperidinol (Cygler *et al.*, 1980), three isomers of (±)-1, 2,3-trimethyl-4-phenyl-4-piperidinol (Cygler & Ahmed, 1984), an anticonvulsant drug, 1-[6-(2-chlorophenyl)-3-pyridazinyl]piperidin-4-ol (Lisgarten & Palmer, 1989), three anticonvulsant compounds, viz., 1-[6-(4-chloro-2-methylphenyl) pyridazin-3-yl]piperidin-4-ol, 1-[6-(4-chlorophenyl)-1,2,4-triazin-3-yl]piperidin-4-ol and 1-[5-(4-methoxyphenyl)pyrimidin-2-yl]piperidin-4-ol (Georges *et al.*, 1989), 1-(4-nitrophenyl)-4-piperidinol (Tomlin *et al.*, 1996), 4-[(*E*)-(2,4-difluorophenyl) (hydroxyimino) methyl]piperidinium picrate (Jasinski *et al.*, 2009), and (4-chlorophenyl)piperidin-4-ol (Dutkiewicz *et al.*, 2010) have been reported. In view of the importance of piperidines, the paper reports the crystal structure of the title compound, (I).

The asymmetric unit of (I) (Fig.1), consists of two crystallographically independent 4-(4-chlorophenyl)-4-hydroxypiperidinium cations (A & B), two picrate anions (A & B) and a dimethylsulfoxide solvent molecule. In the piperidinium cations, bond lengths involving protonated atoms N1A and N1B are 1.507 (2) Å (N1A–C9A), 1.495 (2) Å (N1A–C10A);, 1.492 (3) Å (N1B–C9B) and 1.493 (2) Å (N1B–C10B), which are longer than those found in other structures and the value 1.469 Å given by Allen *et al.*, (1987). In each cation, the piperidine ring adopts a chair conformation with puckering parameters Q = 0.582 (2) Å, Θ = 177.2 (2) ° and φ = 98 (3) ° for molecule A and Q = 0.584 (2) Å, Θ = 0.8 (2) ° and φ = 319 (9) ° for molecule B (Cremer & Pople, 1975).

During crystallization, the removal of the phenolic H atom leads to a shortening of the C12A–O2A = 1.246 (2) Å and C12B–O2B = 1.249 (2) Å bond lengths, indicating partial double bond character. This behaviour is similar to that observed in many picrate salts and is attributed to the loss of the hydroxyl proton at O2A and O2B, leading to the conversion of the neutral to an anionic state of the molecule. This leads to lengthening of the C12A–C13A = 1.454 (2) Å, C12A–C17A = 1.459 (3) Å (molecule A), C12B–C13B = 1.450 (3) Å and C12B–C17B = 1.454 (2) Å (molecule B) bonds compared to the remaining aromatic C–C distances in the picrate ions which has been observed in almost all picrate salts (Anitha *et al.*, 2004; Thanigaimani *et al.*, 2009).

The twist angles of the nitro groups of the each picrate anions shows that the ortho N2A and N4A nitro groups in molecule A and N2B and N4B groups in molecule B deviate from the benzene plane by 30.17 (11), 19.02 (11), 36.53 (11) and 19.73 (12) °, respectively.

In the crystal structure (Fig. 2), cations, anions and solvent molecules are connected by intermolecular N—H···O, O—H···O and C—H···O hydrogen bonds (Table 1), forming a three-dimensional network.

### Experimental

(4-Chlorophenyl)piperidin-4-ol (2.12 g, 0.01 mol) and and picric acid (2.4 g, 0.01 mol) were each dissolved in methanol (25 ml). The solutions were mixed and stirred in a beaker at 323 K for 30 minutes. The mixture was kept aside for three days at room temperature. The formed salt was filtered & dried in vacuum desiccator over phosphorous pentoxide (m.pt. 403-405 K). The salt was recrystallized from dimethylsulfoxide by slow evaporation to yield the title compound.

### Refinement

All hydrogen atoms were positioned geometrically [O–H = 0.82 Å, N–H = 0.90 Å, and C–H = 0.93–0.97 Å] and were refined using a riding model, with *U*_iso_(H) = 1.2 or 1.5 *U*_eq_(C). In the absence of significant anomalous dispersion, 6635 Friedel pairs were merged for the final refinement.

### Crystal data

**Table:**

2C_11_H_15_ClNO^+^·2C_6_H_2_N_3_O_7_^−^·C_2_H_6_OS	*F*(000) = 996
*M**_r_* = 959.72	*D*_x_ = 1.533 Mg m^−^^3^
Monoclinic, *P*2_1_	Mo *K*α radiation, λ = 0.71073 Å
Hall symbol: P 2yb	Cell parameters from 9240 reflections
*a* = 8.9207 (4) Å	θ = 2.6–34.7°
*b* = 18.1230 (9) Å	µ = 0.29 mm^−^^1^
*c* = 12.9886 (6) Å	*T* = 100 K
β = 98.430 (1)°	Block, yellow
*V* = 2077.18 (17) Å^3^	0.40 × 0.32 × 0.16 mm
*Z* = 2	

### Data collection

**Table:**

Bruker APEX DUO CCD area-detector diffractometer	15185 independent reflections
Radiation source: fine-focus sealed tube	13604 reflections with *I* > 2σ(*I*)
graphite	*R*_int_ = 0.029
φ and ω scans	θ_max_ = 34.0°, θ_min_ = 2.3°
Absorption correction: multi-scan (*SADABS*; Bruker, 2009)	*h* = −13→11
*T*_min_ = 0.893, *T*_max_ = 0.954	*k* = −25→28
25314 measured reflections	*l* = −20→20

### Refinement

**Table:**

Refinement on *F*^2^	Primary atom site location: structure-invariant direct methods
Least-squares matrix: full	Secondary atom site location: difference Fourier map
*R*[*F*^2^ > 2σ(*F*^2^)] = 0.041	Hydrogen site location: inferred from neighbouring sites
*wR*(*F*^2^) = 0.140	H-atom parameters constrained
*S* = 1.11	*w* = 1/[σ^2^(*F*_o_^2^) + (0.0893*P*)^2^] where *P* = (*F*_o_^2^ + 2*F*_c_^2^)/3
15185 reflections	(Δ/σ)_max_ < 0.001
582 parameters	Δρ_max_ = 0.83 e Å^−^^3^
1 restraint	Δρ_min_ = −0.88 e Å^−^^3^

### Special details

**Table:**

Experimental. The crystal was placed in the cold stream of an Oxford Cryosystems Cobra open-flow nitrogen cryostat (Cosier & Glazer, 1986) operating at 100.0 (1) K.
Geometry. All s.u.'s (except the s.u. in the dihedral angle between two l.s. planes) are estimated using the full covariance matrix. The cell s.u.'s are taken into account individually in the estimation of s.u.'s in distances, angles and torsion angles; correlations between s.u.'s in cell parameters are only used when they are defined by crystal symmetry. An approximate (isotropic) treatment of cell s.u.'s is used for estimating s.u.'s involving l.s. planes.
Refinement. Refinement of F^2^ against ALL reflections. The weighted R-factor wR and goodness of fit S are based on F^2^, conventional R-factors R are based on F, with F set to zero for negative F^2^. The threshold expression of F^2^ > 2σ(F^2^) is used only for calculating R-factors(gt) etc. and is not relevant to the choice of reflections for refinement. R-factors based on F^2^ are statistically about twice as large as those based on F, and R- factors based on ALL data will be even larger.

### Fractional atomic coordinates and isotropic or equivalent isotropic displacement parameters (Å^2^)

**Table:**

	*x*	*y*	*z*	*U*_iso_*/*U*_eq_	
Cl1A	0.54080 (6)	0.78062 (3)	0.70106 (3)	0.01956 (9)	
O1A	0.61893 (15)	1.07386 (8)	0.41761 (10)	0.0143 (2)	
H1A	0.6671	1.0721	0.4764	0.021*	
N1A	0.66742 (19)	1.05337 (9)	0.15938 (12)	0.0154 (3)	
H1AB	0.6807	1.0893	0.1137	0.019*	
H1AC	0.6638	1.0100	0.1254	0.019*	
C1A	0.4866 (2)	0.89223 (11)	0.43262 (14)	0.0162 (3)	
H1AA	0.4188	0.8926	0.3710	0.019*	
C2A	0.4670 (2)	0.84096 (12)	0.51056 (15)	0.0175 (3)	
H2AA	0.3869	0.8076	0.5012	0.021*	
C3A	0.5690 (2)	0.84107 (11)	0.60158 (14)	0.0141 (3)	
C4A	0.6917 (2)	0.88914 (12)	0.61605 (14)	0.0176 (3)	
H4AA	0.7609	0.8877	0.6770	0.021*	
C5A	0.7090 (2)	0.93908 (11)	0.53828 (14)	0.0167 (3)	
H5AA	0.7911	0.9712	0.5475	0.020*	
C6A	0.60617 (19)	0.94264 (11)	0.44600 (13)	0.0124 (3)	
C7A	0.62592 (19)	1.00352 (10)	0.36771 (13)	0.0112 (3)	
C8A	0.77725 (19)	0.99468 (11)	0.32607 (14)	0.0139 (3)	
H8AA	0.8595	0.9978	0.3837	0.017*	
H8AB	0.7810	0.9462	0.2949	0.017*	
C9A	0.8002 (2)	1.05298 (11)	0.24570 (14)	0.0154 (3)	
H9AA	0.8103	1.1011	0.2786	0.019*	
H9AB	0.8926	1.0427	0.2172	0.019*	
C10A	0.5206 (2)	1.06552 (12)	0.19942 (14)	0.0154 (3)	
H10A	0.4379	1.0655	0.1419	0.018*	
H10B	0.5221	1.1130	0.2339	0.018*	
C11A	0.4964 (2)	1.00446 (11)	0.27584 (13)	0.0138 (3)	
H11A	0.4916	0.9572	0.2405	0.017*	
H11B	0.4008	1.0122	0.3015	0.017*	
Cl1B	0.08958 (5)	0.91889 (3)	0.68165 (4)	0.02007 (9)	
O1B	0.13549 (15)	0.60441 (8)	0.41992 (10)	0.0136 (2)	
H1B	0.0543	0.5928	0.4378	0.020*	
N1B	0.14188 (19)	0.62391 (10)	0.15802 (12)	0.0149 (3)	
H1BB	0.1508	0.5882	0.1111	0.018*	
H1BC	0.1357	0.6675	0.1245	0.018*	
C1B	0.1208 (2)	0.72111 (11)	0.55239 (14)	0.0171 (3)	
H1BA	0.1357	0.6727	0.5755	0.020*	
C2B	0.1142 (2)	0.77734 (12)	0.62472 (14)	0.0179 (3)	
H2BA	0.1244	0.7667	0.6954	0.022*	
C3B	0.0925 (2)	0.84893 (11)	0.59017 (14)	0.0149 (3)	
C4B	0.0750 (2)	0.86606 (11)	0.48428 (14)	0.0161 (3)	
H4BA	0.0601	0.9146	0.4617	0.019*	
C5B	0.0803 (2)	0.80906 (11)	0.41349 (14)	0.0148 (3)	
H5BA	0.0669	0.8197	0.3427	0.018*	
C6B	0.10545 (19)	0.73598 (10)	0.44641 (13)	0.0118 (3)	
C7B	0.12034 (19)	0.67426 (10)	0.36860 (13)	0.0117 (3)	
C8B	0.2656 (2)	0.68375 (11)	0.31946 (14)	0.0141 (3)	
H8BA	0.2647	0.7318	0.2864	0.017*	
H8BB	0.3529	0.6817	0.3735	0.017*	
C9B	0.2793 (2)	0.62363 (11)	0.23875 (15)	0.0160 (3)	
H9BA	0.3688	0.6323	0.2062	0.019*	
H9BB	0.2898	0.5758	0.2727	0.019*	
C10B	0.0000 (2)	0.61169 (11)	0.20400 (14)	0.0147 (3)	
H10C	0.0040	0.5639	0.2380	0.018*	
H10D	−0.0866	0.6120	0.1493	0.018*	
C11B	−0.0179 (2)	0.67201 (11)	0.28259 (14)	0.0133 (3)	
H11C	−0.1088	0.6630	0.3135	0.016*	
H11D	−0.0290	0.7194	0.2474	0.016*	
O2A	0.61771 (16)	0.91428 (8)	0.08706 (11)	0.0167 (2)	
O3A	0.7941 (2)	0.99873 (9)	−0.01439 (12)	0.0238 (3)	
O4A	0.8540 (2)	0.95285 (9)	−0.15570 (12)	0.0232 (3)	
O5A	0.9570 (2)	0.69858 (10)	−0.17516 (15)	0.0323 (4)	
O6A	0.8761 (2)	0.62139 (10)	−0.06713 (16)	0.0306 (4)	
O7A	0.6214 (2)	0.71007 (10)	0.20625 (14)	0.0265 (3)	
O8A	0.47444 (18)	0.80551 (10)	0.17469 (13)	0.0243 (3)	
N2A	0.80695 (19)	0.94537 (9)	−0.07213 (12)	0.0148 (3)	
N3A	0.8834 (2)	0.68406 (10)	−0.10369 (15)	0.0206 (3)	
N4A	0.57910 (18)	0.76640 (10)	0.15561 (13)	0.0162 (3)	
C12A	0.67605 (19)	0.86314 (10)	0.04248 (13)	0.0125 (3)	
C13A	0.76790 (19)	0.87243 (10)	−0.04033 (13)	0.0123 (3)	
C14A	0.8299 (2)	0.81494 (10)	−0.09002 (13)	0.0131 (3)	
H14A	0.8853	0.8242	−0.1440	0.016*	
C15A	0.8079 (2)	0.74346 (11)	−0.05797 (14)	0.0145 (3)	
C16A	0.7243 (2)	0.72826 (11)	0.02189 (14)	0.0147 (3)	
H16A	0.7119	0.6799	0.0430	0.018*	
C17A	0.66026 (19)	0.78560 (11)	0.06925 (13)	0.0132 (3)	
O2B	0.11214 (17)	0.76690 (8)	0.09635 (11)	0.0175 (3)	
O3B	−0.03236 (16)	0.88222 (9)	0.17146 (11)	0.0191 (3)	
O4B	0.1375 (2)	0.96634 (11)	0.21473 (14)	0.0286 (4)	
O5B	0.3832 (2)	1.05229 (9)	−0.07138 (16)	0.0295 (4)	
O6B	0.47519 (19)	0.97024 (9)	−0.16764 (13)	0.0230 (3)	
O7B	0.3277 (2)	0.72048 (9)	−0.15469 (13)	0.0277 (4)	
O8B	0.2714 (3)	0.67703 (10)	−0.01198 (14)	0.0320 (4)	
N2B	0.08429 (17)	0.91522 (10)	0.15888 (12)	0.0145 (3)	
N3B	0.39467 (19)	0.98818 (10)	−0.10184 (13)	0.0170 (3)	
N4B	0.2868 (2)	0.72954 (10)	−0.06939 (12)	0.0157 (3)	
C12B	0.17235 (19)	0.81592 (10)	0.04862 (13)	0.0127 (3)	
C13B	0.16278 (19)	0.89373 (11)	0.07279 (13)	0.0123 (3)	
C14B	0.23231 (19)	0.94922 (11)	0.02524 (14)	0.0141 (3)	
H14B	0.2259	0.9979	0.0466	0.017*	
C15B	0.3126 (2)	0.93112 (10)	−0.05568 (14)	0.0135 (3)	
C16B	0.32577 (19)	0.85917 (11)	−0.08763 (13)	0.0135 (3)	
H16B	0.3788	0.8480	−0.1422	0.016*	
C17B	0.2583 (2)	0.80354 (10)	−0.03668 (14)	0.0129 (3)	
S1	0.74330 (6)	0.56505 (3)	0.52501 (4)	0.02135 (10)	
O9	0.90638 (17)	0.55756 (10)	0.50845 (13)	0.0231 (3)	
C18	0.7073 (3)	0.66185 (18)	0.5327 (3)	0.0446 (8)	
H18A	0.7794	0.6834	0.5865	0.067*	
H18B	0.6067	0.6694	0.5486	0.067*	
H18C	0.7166	0.6847	0.4672	0.067*	
C19	0.6293 (2)	0.54900 (15)	0.40268 (18)	0.0258 (4)	
H19A	0.6479	0.5001	0.3791	0.039*	
H19B	0.6544	0.5844	0.3529	0.039*	
H19C	0.5243	0.5539	0.4101	0.039*	

### Atomic displacement parameters (Å^2^)

**Table:**

	*U*^11^	*U*^22^	*U*^33^	*U*^12^	*U*^13^	*U*^23^
Cl1A	0.0259 (2)	0.0181 (2)	0.01581 (17)	−0.00290 (17)	0.00703 (15)	0.00415 (16)
O1A	0.0171 (5)	0.0120 (6)	0.0136 (5)	−0.0012 (4)	0.0020 (4)	−0.0027 (4)
N1A	0.0225 (7)	0.0125 (7)	0.0123 (6)	−0.0006 (5)	0.0061 (5)	0.0002 (5)
C1A	0.0156 (7)	0.0174 (8)	0.0153 (7)	−0.0051 (6)	0.0016 (6)	0.0013 (6)
C2A	0.0166 (7)	0.0186 (9)	0.0172 (7)	−0.0067 (6)	0.0023 (6)	0.0021 (6)
C3A	0.0172 (7)	0.0135 (8)	0.0124 (6)	−0.0003 (6)	0.0050 (5)	0.0022 (6)
C4A	0.0206 (7)	0.0175 (9)	0.0139 (7)	−0.0032 (6)	−0.0008 (6)	0.0015 (6)
C5A	0.0171 (7)	0.0152 (8)	0.0167 (7)	−0.0053 (6)	−0.0006 (6)	0.0019 (6)
C6A	0.0132 (6)	0.0120 (7)	0.0125 (6)	−0.0017 (5)	0.0033 (5)	−0.0002 (6)
C7A	0.0123 (6)	0.0090 (7)	0.0123 (6)	−0.0007 (5)	0.0023 (5)	−0.0013 (5)
C8A	0.0144 (7)	0.0126 (8)	0.0154 (7)	0.0006 (6)	0.0044 (5)	0.0013 (6)
C9A	0.0163 (7)	0.0142 (8)	0.0170 (7)	−0.0014 (6)	0.0065 (6)	−0.0006 (6)
C10A	0.0186 (7)	0.0140 (8)	0.0137 (7)	0.0006 (6)	0.0031 (6)	0.0011 (6)
C11A	0.0147 (6)	0.0145 (8)	0.0121 (6)	−0.0021 (6)	0.0016 (5)	−0.0006 (6)
Cl1B	0.02299 (19)	0.0197 (2)	0.01767 (18)	0.00048 (16)	0.00359 (15)	−0.00778 (16)
O1B	0.0165 (5)	0.0100 (6)	0.0146 (5)	0.0016 (4)	0.0036 (4)	0.0029 (4)
N1B	0.0226 (7)	0.0115 (7)	0.0120 (6)	−0.0004 (5)	0.0069 (5)	−0.0014 (5)
C1B	0.0264 (8)	0.0142 (8)	0.0107 (6)	0.0005 (6)	0.0032 (6)	0.0010 (6)
C2B	0.0252 (8)	0.0173 (9)	0.0118 (7)	0.0010 (7)	0.0043 (6)	−0.0001 (6)
C3B	0.0157 (7)	0.0147 (8)	0.0146 (7)	−0.0004 (6)	0.0030 (5)	−0.0041 (6)
C4B	0.0197 (7)	0.0131 (8)	0.0153 (7)	0.0027 (6)	0.0015 (6)	−0.0001 (6)
C5B	0.0195 (7)	0.0133 (8)	0.0116 (6)	0.0029 (6)	0.0018 (6)	0.0011 (6)
C6B	0.0131 (6)	0.0113 (7)	0.0109 (6)	0.0006 (5)	0.0020 (5)	0.0003 (5)
C7B	0.0144 (6)	0.0096 (7)	0.0112 (6)	0.0003 (5)	0.0026 (5)	0.0012 (5)
C8B	0.0143 (6)	0.0129 (8)	0.0158 (7)	−0.0001 (6)	0.0051 (6)	−0.0005 (6)
C9B	0.0176 (7)	0.0151 (8)	0.0170 (7)	0.0005 (6)	0.0079 (6)	−0.0010 (6)
C10B	0.0177 (7)	0.0141 (8)	0.0123 (6)	−0.0020 (6)	0.0026 (5)	−0.0003 (6)
C11B	0.0141 (7)	0.0118 (8)	0.0138 (7)	0.0000 (6)	0.0011 (5)	−0.0005 (6)
O2A	0.0194 (6)	0.0141 (6)	0.0181 (6)	−0.0003 (5)	0.0073 (5)	−0.0033 (5)
O3A	0.0447 (9)	0.0107 (7)	0.0189 (6)	−0.0030 (6)	0.0144 (6)	−0.0030 (5)
O4A	0.0412 (9)	0.0140 (7)	0.0179 (6)	−0.0003 (6)	0.0166 (6)	0.0008 (5)
O5A	0.0469 (11)	0.0186 (8)	0.0383 (9)	0.0048 (7)	0.0293 (9)	0.0016 (7)
O6A	0.0434 (10)	0.0113 (7)	0.0423 (10)	0.0027 (7)	0.0233 (8)	0.0029 (7)
O7A	0.0287 (7)	0.0238 (8)	0.0286 (8)	0.0038 (6)	0.0097 (6)	0.0132 (6)
O8A	0.0204 (6)	0.0275 (8)	0.0269 (7)	0.0030 (6)	0.0104 (6)	0.0050 (6)
N2A	0.0209 (7)	0.0109 (7)	0.0129 (6)	0.0001 (5)	0.0040 (5)	0.0013 (5)
N3A	0.0262 (8)	0.0112 (8)	0.0273 (8)	0.0017 (6)	0.0134 (7)	0.0002 (6)
N4A	0.0150 (6)	0.0165 (8)	0.0172 (7)	−0.0035 (5)	0.0030 (5)	0.0021 (5)
C12A	0.0131 (6)	0.0126 (8)	0.0119 (6)	−0.0006 (5)	0.0017 (5)	−0.0007 (6)
C13A	0.0160 (7)	0.0082 (7)	0.0126 (6)	0.0006 (5)	0.0021 (5)	0.0008 (5)
C14A	0.0160 (7)	0.0121 (8)	0.0117 (6)	0.0000 (6)	0.0036 (5)	0.0000 (6)
C15A	0.0174 (7)	0.0098 (8)	0.0169 (7)	0.0007 (6)	0.0047 (6)	−0.0012 (6)
C16A	0.0154 (7)	0.0111 (8)	0.0178 (7)	−0.0008 (6)	0.0034 (6)	0.0019 (6)
C17A	0.0135 (6)	0.0128 (8)	0.0136 (6)	−0.0008 (6)	0.0028 (5)	0.0019 (6)
O2B	0.0205 (6)	0.0141 (7)	0.0196 (6)	0.0002 (5)	0.0090 (5)	0.0018 (5)
O3B	0.0157 (5)	0.0243 (8)	0.0185 (6)	−0.0011 (5)	0.0065 (5)	−0.0018 (5)
O4B	0.0245 (7)	0.0317 (10)	0.0308 (8)	−0.0068 (6)	0.0081 (6)	−0.0191 (7)
O5B	0.0408 (9)	0.0094 (7)	0.0430 (10)	−0.0026 (6)	0.0213 (8)	−0.0013 (6)
O6B	0.0284 (7)	0.0187 (7)	0.0247 (7)	−0.0026 (6)	0.0131 (6)	0.0006 (6)
O7B	0.0472 (10)	0.0157 (7)	0.0261 (7)	−0.0023 (7)	0.0250 (7)	−0.0028 (6)
O8B	0.0656 (13)	0.0120 (7)	0.0245 (7)	0.0037 (7)	0.0269 (8)	0.0035 (6)
N2B	0.0133 (6)	0.0169 (8)	0.0133 (6)	0.0024 (5)	0.0014 (5)	−0.0020 (6)
N3B	0.0186 (6)	0.0126 (7)	0.0208 (7)	−0.0013 (5)	0.0058 (5)	0.0025 (6)
N4B	0.0240 (7)	0.0115 (7)	0.0122 (6)	−0.0007 (5)	0.0050 (5)	0.0003 (5)
C12B	0.0139 (6)	0.0122 (8)	0.0119 (6)	0.0006 (5)	0.0016 (5)	−0.0004 (6)
C13B	0.0121 (6)	0.0128 (7)	0.0123 (6)	0.0015 (5)	0.0025 (5)	−0.0007 (6)
C14B	0.0136 (6)	0.0121 (8)	0.0167 (7)	0.0010 (5)	0.0022 (6)	0.0003 (6)
C15B	0.0144 (6)	0.0106 (8)	0.0157 (7)	−0.0005 (5)	0.0032 (5)	0.0015 (6)
C16B	0.0152 (7)	0.0125 (8)	0.0128 (7)	0.0005 (6)	0.0024 (5)	0.0013 (6)
C17B	0.0165 (7)	0.0097 (7)	0.0130 (7)	0.0010 (6)	0.0035 (5)	−0.0004 (5)
S1	0.01909 (19)	0.0257 (3)	0.0210 (2)	−0.00352 (17)	0.00883 (16)	0.00180 (18)
O9	0.0174 (6)	0.0231 (8)	0.0303 (7)	−0.0003 (5)	0.0083 (5)	0.0068 (6)
C18	0.0301 (12)	0.0316 (15)	0.077 (2)	−0.0032 (10)	0.0228 (13)	−0.0224 (15)
C19	0.0195 (8)	0.0338 (13)	0.0250 (9)	0.0008 (8)	0.0064 (7)	0.0009 (8)

### Geometric parameters (Å, °)

**Table:**

Cl1A—C3A	1.7402 (18)	C9B—H9BA	0.9700
O1A—C7A	1.436 (2)	C9B—H9BB	0.9700
O1A—H1A	0.8200	C10B—C11B	1.520 (3)
N1A—C10A	1.495 (2)	C10B—H10C	0.9700
N1A—C9A	1.507 (2)	C10B—H10D	0.9700
N1A—H1AB	0.9000	C11B—H11C	0.9700
N1A—H1AC	0.9000	C11B—H11D	0.9700
C1A—C6A	1.396 (2)	O2A—C12A	1.246 (2)
C1A—C2A	1.404 (3)	O3A—N2A	1.239 (2)
C1A—H1AA	0.9300	O4A—N2A	1.227 (2)
C2A—C3A	1.383 (3)	O5A—N3A	1.241 (2)
C2A—H2AA	0.9300	O6A—N3A	1.236 (2)
C3A—C4A	1.390 (3)	O7A—N4A	1.243 (2)
C4A—C5A	1.382 (3)	O8A—N4A	1.226 (2)
C4A—H4AA	0.9300	N2A—C13A	1.443 (3)
C5A—C6A	1.400 (2)	N3A—C15A	1.443 (3)
C5A—H5AA	0.9300	N4A—C17A	1.463 (2)
C6A—C7A	1.528 (2)	C12A—C13A	1.454 (2)
C7A—C8A	1.534 (2)	C12A—C17A	1.459 (3)
C7A—C11A	1.535 (2)	C13A—C14A	1.383 (3)
C8A—C9A	1.520 (3)	C14A—C15A	1.383 (3)
C8A—H8AA	0.9700	C14A—H14A	0.9300
C8A—H8AB	0.9700	C15A—C16A	1.391 (3)
C9A—H9AA	0.9700	C16A—C17A	1.374 (3)
C9A—H9AB	0.9700	C16A—H16A	0.9300
C10A—C11A	1.523 (3)	O2B—C12B	1.249 (2)
C10A—H10A	0.9700	O3B—N2B	1.232 (2)
C10A—H10B	0.9700	O4B—N2B	1.228 (2)
C11A—H11A	0.9700	O5B—N3B	1.236 (2)
C11A—H11B	0.9700	O6B—N3B	1.238 (2)
Cl1B—C3B	1.7405 (19)	O7B—N4B	1.227 (2)
O1B—C7B	1.428 (2)	O8B—N4B	1.229 (2)
O1B—H1B	0.8200	N2B—C13B	1.457 (2)
N1B—C9B	1.492 (3)	N3B—C15B	1.447 (2)
N1B—C10B	1.493 (2)	N4B—C17B	1.441 (3)
N1B—H1BB	0.9000	C12B—C13B	1.450 (3)
N1B—H1BC	0.9000	C12B—C17B	1.454 (2)
C1B—C6B	1.390 (2)	C13B—C14B	1.375 (3)
C1B—C2B	1.393 (3)	C14B—C15B	1.395 (3)
C1B—H1BA	0.9300	C14B—H14B	0.9300
C2B—C3B	1.377 (3)	C15B—C16B	1.379 (3)
C2B—H2BA	0.9300	C16B—C17B	1.391 (3)
C3B—C4B	1.396 (3)	C16B—H16B	0.9300
C4B—C5B	1.388 (3)	S1—O9	1.5076 (16)
C4B—H4BA	0.9300	S1—C19	1.780 (2)
C5B—C6B	1.400 (3)	S1—C18	1.789 (3)
C5B—H5BA	0.9300	C18—H18A	0.9600
C6B—C7B	1.526 (2)	C18—H18B	0.9600
C7B—C8B	1.536 (2)	C18—H18C	0.9600
C7B—C11B	1.538 (2)	C19—H19A	0.9600
C8B—C9B	1.529 (3)	C19—H19B	0.9600
C8B—H8BA	0.9700	C19—H19C	0.9600
C8B—H8BB	0.9700		
			
C7A—O1A—H1A	109.5	C7B—C8B—H8BB	109.4
C10A—N1A—C9A	112.02 (14)	H8BA—C8B—H8BB	108.0
C10A—N1A—H1AB	109.2	N1B—C9B—C8B	109.85 (15)
C9A—N1A—H1AB	109.2	N1B—C9B—H9BA	109.7
C10A—N1A—H1AC	109.2	C8B—C9B—H9BA	109.7
C9A—N1A—H1AC	109.2	N1B—C9B—H9BB	109.7
H1AB—N1A—H1AC	107.9	C8B—C9B—H9BB	109.7
C6A—C1A—C2A	121.15 (17)	H9BA—C9B—H9BB	108.2
C6A—C1A—H1AA	119.4	N1B—C10B—C11B	109.90 (15)
C2A—C1A—H1AA	119.4	N1B—C10B—H10C	109.7
C3A—C2A—C1A	118.65 (17)	C11B—C10B—H10C	109.7
C3A—C2A—H2AA	120.7	N1B—C10B—H10D	109.7
C1A—C2A—H2AA	120.7	C11B—C10B—H10D	109.7
C2A—C3A—C4A	121.56 (17)	H10C—C10B—H10D	108.2
C2A—C3A—Cl1A	119.02 (14)	C10B—C11B—C7B	110.79 (15)
C4A—C3A—Cl1A	119.41 (14)	C10B—C11B—H11C	109.5
C5A—C4A—C3A	118.80 (17)	C7B—C11B—H11C	109.5
C5A—C4A—H4AA	120.6	C10B—C11B—H11D	109.5
C3A—C4A—H4AA	120.6	C7B—C11B—H11D	109.5
C4A—C5A—C6A	121.80 (17)	H11C—C11B—H11D	108.1
C4A—C5A—H5AA	119.1	O4A—N2A—O3A	121.54 (17)
C6A—C5A—H5AA	119.1	O4A—N2A—C13A	118.85 (16)
C1A—C6A—C5A	117.99 (16)	O3A—N2A—C13A	119.60 (15)
C1A—C6A—C7A	123.39 (16)	O6A—N3A—O5A	123.05 (19)
C5A—C6A—C7A	118.56 (16)	O6A—N3A—C15A	118.24 (17)
O1A—C7A—C6A	108.89 (13)	O5A—N3A—C15A	118.66 (18)
O1A—C7A—C8A	110.52 (14)	O8A—N4A—O7A	123.30 (17)
C6A—C7A—C8A	110.63 (14)	O8A—N4A—C17A	119.63 (17)
O1A—C7A—C11A	105.28 (14)	O7A—N4A—C17A	117.07 (17)
C6A—C7A—C11A	112.23 (14)	O2A—C12A—C13A	125.20 (17)
C8A—C7A—C11A	109.16 (14)	O2A—C12A—C17A	122.95 (16)
C9A—C8A—C7A	112.54 (15)	C13A—C12A—C17A	111.85 (15)
C9A—C8A—H8AA	109.1	C14A—C13A—N2A	115.26 (15)
C7A—C8A—H8AA	109.1	C14A—C13A—C12A	124.41 (17)
C9A—C8A—H8AB	109.1	N2A—C13A—C12A	120.24 (16)
C7A—C8A—H8AB	109.1	C13A—C14A—C15A	118.76 (16)
H8AA—C8A—H8AB	107.8	C13A—C14A—H14A	120.6
N1A—C9A—C8A	110.14 (15)	C15A—C14A—H14A	120.6
N1A—C9A—H9AA	109.6	C14A—C15A—C16A	121.66 (17)
C8A—C9A—H9AA	109.6	C14A—C15A—N3A	118.66 (16)
N1A—C9A—H9AB	109.6	C16A—C15A—N3A	119.52 (17)
C8A—C9A—H9AB	109.6	C17A—C16A—C15A	119.20 (17)
H9AA—C9A—H9AB	108.1	C17A—C16A—H16A	120.4
N1A—C10A—C11A	109.57 (16)	C15A—C16A—H16A	120.4
N1A—C10A—H10A	109.8	C16A—C17A—C12A	124.09 (16)
C11A—C10A—H10A	109.8	C16A—C17A—N4A	116.64 (17)
N1A—C10A—H10B	109.8	C12A—C17A—N4A	119.17 (16)
C11A—C10A—H10B	109.8	O4B—N2B—O3B	123.28 (17)
H10A—C10A—H10B	108.2	O4B—N2B—C13B	117.86 (16)
C10A—C11A—C7A	110.63 (15)	O3B—N2B—C13B	118.85 (16)
C10A—C11A—H11A	109.5	O5B—N3B—O6B	123.47 (18)
C7A—C11A—H11A	109.5	O5B—N3B—C15B	117.93 (17)
C10A—C11A—H11B	109.5	O6B—N3B—C15B	118.58 (17)
C7A—C11A—H11B	109.5	O7B—N4B—O8B	121.23 (18)
H11A—C11A—H11B	108.1	O7B—N4B—C17B	118.65 (16)
C7B—O1B—H1B	109.5	O8B—N4B—C17B	120.11 (15)
C9B—N1B—C10B	112.08 (14)	O2B—C12B—C13B	122.76 (16)
C9B—N1B—H1BB	109.2	O2B—C12B—C17B	125.56 (17)
C10B—N1B—H1BB	109.2	C13B—C12B—C17B	111.68 (15)
C9B—N1B—H1BC	109.2	C14B—C13B—C12B	124.71 (16)
C10B—N1B—H1BC	109.2	C14B—C13B—N2B	116.66 (17)
H1BB—N1B—H1BC	107.9	C12B—C13B—N2B	118.45 (16)
C6B—C1B—C2B	121.22 (18)	C13B—C14B—C15B	118.81 (17)
C6B—C1B—H1BA	119.4	C13B—C14B—H14B	120.6
C2B—C1B—H1BA	119.4	C15B—C14B—H14B	120.6
C3B—C2B—C1B	119.16 (17)	C16B—C15B—C14B	121.65 (17)
C3B—C2B—H2BA	120.4	C16B—C15B—N3B	118.75 (16)
C1B—C2B—H2BA	120.4	C14B—C15B—N3B	119.41 (17)
C2B—C3B—C4B	121.36 (17)	C15B—C16B—C17B	118.73 (16)
C2B—C3B—Cl1B	118.64 (14)	C15B—C16B—H16B	120.6
C4B—C3B—Cl1B	119.99 (15)	C17B—C16B—H16B	120.6
C5B—C4B—C3B	118.51 (18)	C16B—C17B—N4B	115.28 (15)
C5B—C4B—H4BA	120.7	C16B—C17B—C12B	124.34 (17)
C3B—C4B—H4BA	120.7	N4B—C17B—C12B	120.27 (16)
C4B—C5B—C6B	121.41 (16)	O9—S1—C19	107.14 (10)
C4B—C5B—H5BA	119.3	O9—S1—C18	106.33 (12)
C6B—C5B—H5BA	119.3	C19—S1—C18	97.27 (15)
C1B—C6B—C5B	118.31 (17)	S1—C18—H18A	109.5
C1B—C6B—C7B	120.55 (16)	S1—C18—H18B	109.5
C5B—C6B—C7B	121.12 (15)	H18A—C18—H18B	109.5
O1B—C7B—C6B	110.62 (14)	S1—C18—H18C	109.5
O1B—C7B—C8B	105.39 (14)	H18A—C18—H18C	109.5
C6B—C7B—C8B	110.72 (14)	H18B—C18—H18C	109.5
O1B—C7B—C11B	109.05 (14)	S1—C19—H19A	109.5
C6B—C7B—C11B	111.22 (14)	S1—C19—H19B	109.5
C8B—C7B—C11B	109.66 (14)	H19A—C19—H19B	109.5
C9B—C8B—C7B	111.24 (15)	S1—C19—H19C	109.5
C9B—C8B—H8BA	109.4	H19A—C19—H19C	109.5
C7B—C8B—H8BA	109.4	H19B—C19—H19C	109.5
C9B—C8B—H8BB	109.4		
			
C6A—C1A—C2A—C3A	−0.1 (3)	O4A—N2A—C13A—C12A	−163.82 (17)
C1A—C2A—C3A—C4A	−1.7 (3)	O3A—N2A—C13A—C12A	17.0 (3)
C1A—C2A—C3A—Cl1A	177.09 (15)	O2A—C12A—C13A—C14A	−178.98 (18)
C2A—C3A—C4A—C5A	1.7 (3)	C17A—C12A—C13A—C14A	1.6 (2)
Cl1A—C3A—C4A—C5A	−177.14 (16)	O2A—C12A—C13A—N2A	4.4 (3)
C3A—C4A—C5A—C6A	0.3 (3)	C17A—C12A—C13A—N2A	−175.00 (15)
C2A—C1A—C6A—C5A	2.0 (3)	N2A—C13A—C14A—C15A	174.83 (16)
C2A—C1A—C6A—C7A	−175.00 (18)	C12A—C13A—C14A—C15A	−1.9 (3)
C4A—C5A—C6A—C1A	−2.1 (3)	C13A—C14A—C15A—C16A	0.6 (3)
C4A—C5A—C6A—C7A	175.07 (18)	C13A—C14A—C15A—N3A	−174.74 (17)
C1A—C6A—C7A—O1A	118.45 (19)	O6A—N3A—C15A—C14A	172.6 (2)
C5A—C6A—C7A—O1A	−58.5 (2)	O5A—N3A—C15A—C14A	−4.8 (3)
C1A—C6A—C7A—C8A	−119.90 (19)	O6A—N3A—C15A—C16A	−2.9 (3)
C5A—C6A—C7A—C8A	63.1 (2)	O5A—N3A—C15A—C16A	179.7 (2)
C1A—C6A—C7A—C11A	2.3 (2)	C14A—C15A—C16A—C17A	0.9 (3)
C5A—C6A—C7A—C11A	−174.66 (16)	N3A—C15A—C16A—C17A	176.16 (17)
O1A—C7A—C8A—C9A	−60.77 (19)	C15A—C16A—C17A—C12A	−1.1 (3)
C6A—C7A—C8A—C9A	178.54 (15)	C15A—C16A—C17A—N4A	−177.49 (16)
C11A—C7A—C8A—C9A	54.6 (2)	O2A—C12A—C17A—C16A	−179.49 (18)
C10A—N1A—C9A—C8A	56.6 (2)	C13A—C12A—C17A—C16A	0.0 (2)
C7A—C8A—C9A—N1A	−54.3 (2)	O2A—C12A—C17A—N4A	−3.2 (3)
C9A—N1A—C10A—C11A	−59.5 (2)	C13A—C12A—C17A—N4A	176.24 (15)
N1A—C10A—C11A—C7A	59.51 (19)	O8A—N4A—C17A—C16A	−151.85 (18)
O1A—C7A—C11A—C10A	61.98 (18)	O7A—N4A—C17A—C16A	28.1 (2)
C6A—C7A—C11A—C10A	−179.71 (15)	O8A—N4A—C17A—C12A	31.6 (2)
C8A—C7A—C11A—C10A	−56.7 (2)	O7A—N4A—C17A—C12A	−148.48 (18)
C6B—C1B—C2B—C3B	−0.2 (3)	O2B—C12B—C13B—C14B	177.23 (18)
C1B—C2B—C3B—C4B	0.8 (3)	C17B—C12B—C13B—C14B	−3.2 (2)
C1B—C2B—C3B—Cl1B	−178.29 (15)	O2B—C12B—C13B—N2B	2.3 (3)
C2B—C3B—C4B—C5B	−0.2 (3)	C17B—C12B—C13B—N2B	−178.07 (14)
Cl1B—C3B—C4B—C5B	178.96 (14)	O4B—N2B—C13B—C14B	−33.8 (2)
C3B—C4B—C5B—C6B	−1.2 (3)	O3B—N2B—C13B—C14B	145.35 (18)
C2B—C1B—C6B—C5B	−1.1 (3)	O4B—N2B—C13B—C12B	141.56 (19)
C2B—C1B—C6B—C7B	177.12 (17)	O3B—N2B—C13B—C12B	−39.3 (2)
C4B—C5B—C6B—C1B	1.8 (3)	C12B—C13B—C14B—C15B	3.2 (3)
C4B—C5B—C6B—C7B	−176.40 (16)	N2B—C13B—C14B—C15B	178.19 (15)
C1B—C6B—C7B—O1B	5.6 (2)	C13B—C14B—C15B—C16B	−1.1 (3)
C5B—C6B—C7B—O1B	−176.22 (15)	C13B—C14B—C15B—N3B	−175.95 (16)
C1B—C6B—C7B—C8B	−110.87 (19)	O5B—N3B—C15B—C16B	−179.11 (19)
C5B—C6B—C7B—C8B	67.3 (2)	O6B—N3B—C15B—C16B	−0.8 (3)
C1B—C6B—C7B—C11B	126.93 (18)	O5B—N3B—C15B—C14B	−4.1 (3)
C5B—C6B—C7B—C11B	−54.9 (2)	O6B—N3B—C15B—C14B	174.24 (18)
O1B—C7B—C8B—C9B	62.18 (18)	C14B—C15B—C16B—C17B	−0.7 (3)
C6B—C7B—C8B—C9B	−178.17 (15)	N3B—C15B—C16B—C17B	174.20 (16)
C11B—C7B—C8B—C9B	−55.1 (2)	C15B—C16B—C17B—N4B	−175.51 (16)
C10B—N1B—C9B—C8B	−58.6 (2)	C15B—C16B—C17B—C12B	0.5 (3)
C7B—C8B—C9B—N1B	56.3 (2)	O7B—N4B—C17B—C16B	−20.3 (3)
C9B—N1B—C10B—C11B	59.6 (2)	O8B—N4B—C17B—C16B	159.0 (2)
N1B—C10B—C11B—C7B	−57.6 (2)	O7B—N4B—C17B—C12B	163.47 (18)
O1B—C7B—C11B—C10B	−59.23 (19)	O8B—N4B—C17B—C12B	−17.2 (3)
C6B—C7B—C11B—C10B	178.51 (14)	O2B—C12B—C17B—C16B	−179.16 (18)
C8B—C7B—C11B—C10B	55.7 (2)	C13B—C12B—C17B—C16B	1.2 (2)
O4A—N2A—C13A—C14A	19.3 (2)	O2B—C12B—C17B—N4B	−3.3 (3)
O3A—N2A—C13A—C14A	−159.87 (18)	C13B—C12B—C17B—N4B	177.10 (16)

### Hydrogen-bond geometry (Å, °)

**Table:**

*D*—H···*A*	*D*—H	H···*A*	*D*···*A*	*D*—H···*A*
N1A—H1AB···O7B^i^	0.90	2.44	3.030 (2)	123
N1A—H1AB···O8B^i^	0.90	2.15	3.048 (2)	176
O1A—H1A···O1B^ii^	0.82	2.14	2.8642 (19)	148
O1B—H1B···O9^iii^	0.82	1.83	2.629 (2)	165
N1A—H1AC···O2A	0.90	1.83	2.704 (2)	162
N1A—H1AC···O3A	0.90	2.30	2.846 (2)	119
N1B—H1BB···O3A^iv^	0.90	2.15	3.044 (2)	171
N1B—H1BB···O4A^iv^	0.90	2.52	3.101 (2)	123
N1B—H1BC···O2B	0.90	1.84	2.714 (2)	162
C4A—H4AA···O4A^v^	0.93	2.51	3.311 (2)	145
C5A—H5AA···O1B^ii^	0.93	2.52	3.313 (2)	143
C8A—H8AA···O9^vi^	0.97	2.58	3.479 (2)	155
C9A—H9AB···O4B^vii^	0.97	2.59	3.469 (3)	151
C11A—H11A···O2A	0.97	2.55	3.261 (2)	130
C11A—H11B···O4B	0.97	2.59	3.258 (3)	126
C2B—H2BA···O7B^v^	0.93	2.60	3.361 (3)	140
C14B—H14B···O6A^i^	0.93	2.45	3.335 (3)	160
C5B—H5BA···O3B	0.93	2.54	3.424 (2)	160
C16A—H16A···O5B^iv^	0.93	2.51	3.418 (3)	166

Symmetry codes: (i) −*x*+1, *y*+1/2, −*z*; (ii) −*x*+1, *y*+1/2, −*z*+1; (iii) *x*−1, *y*, *z*; (iv) −*x*+1, *y*−1/2, −*z*; (v) *x*, *y*, *z*+1; (vi) −*x*+2, *y*+1/2, −*z*+1; (vii) *x*+1, *y*, *z*.
